# Photoreceptor Characteristics in Diabetic Retinopathy vs Controls Using Adaptive Optics Imaging: Systematic Review

**DOI:** 10.1177/24741264241286682

**Published:** 2024-09-30

**Authors:** Justin Grad, Amin Hatamnejad, Niveditha Pattathil, John Golding, Netan Choudhry

**Affiliations:** 1 Michael DeGroote School of Medicine, McMaster University, Hamilton, ON, Canada; 2 Vitreous Retina Macula Specialists of Toronto, Etobicoke, ON, Canada; 3 Department of Ophthalmology and Vision Sciences, University of Toronto, Toronto, ON, Canada; 4 Cleveland Clinic Canada, Toronto, ON, Canada; 5 Retina Consultants of Texas, Blanton Eye Institute, Houston Methodist Hospital, Houston, TX, USA

**Keywords:** diabetic retinopathy, ophthalmoscopy, optical coherence tomography, retinal cone photoreceptor cells

## Abstract

**Purpose:** To assess the differences in morphological photoreceptor outcomes measured using adaptive optics (AO)–assisted imaging between individuals with diabetes or prediabetes and healthy controls. **Methods:** A systematic search was conducted across MEDLINE, Embase, and Cochrane databases from January 2000 to June 2023. Studies that used AO-assisted imaging modalities to quantitatively compare photoreceptor outcomes in patients with diabetes or prediabetes with healthy controls were included. **Results**: Eleven studies consisting of 551 eyes were included. Most studies reported significant differences in photoreceptor outcomes between diabetic and healthy populations, particularly as diabetic retinopathy (DR) severity increased. Cone regularity was the most sensitive parameter for detecting significant differences between groups. AO imaging was less reliable in distinguishing individuals with diabetes without DR or with mild DR severity from controls. **Conclusions:** AO imaging showed promise in detecting significant differences associated with diabetes and DR, in particular with increasing disease severity. Further research is warranted to assess AO’s utility as a diabetes and DR screening tool. Standardizing imaging protocols in future studies is recommended to allow for more direct quantitative comparisons. These findings highlight the current evidence on photoreceptor changes in patients with diabetes and the potential of AO in advancing diabetic eye care.

## Introduction

Diabetes mellitus is a complex medical condition characterized by inadequate control of blood glucose levels and serious systemic complications including nephropathy, retinopathy, neuropathy, and atherosclerotic cardiovascular disease.^1^ In addition, diabetes has serious economic repercussions. In the United States, for instance, the cost of medical care and reduced productivity due to diabetes was estimated to be $327 billion in 2017.^2^ The prevalence of diabetes is increasing globally, and the number of individuals with diabetes is expected to increase from an estimated 540 million in 2021 to nearly 800 million by 2045.^3^

Patients with diabetes are monitored for the progression of diabetic retinopathy (DR), a serious ocular condition that can lead to progressive vision loss and in some cases blindness.^4^ A significant association has been found between blood glucose levels and the development of DR, emphasizing the importance of adequate blood glucose control.^5^ The early detection of diabetes is crucial to improving future outcomes because early pharmacotherapy intervention and patient education lead to better blood glucose control.^6^

In patients whose diabetes was diagnosed through asymptomatic screening, the prevalence and severity of DR was shown to be significantly lower than in those whose diabetes was diagnosed through conventional care.^7^ Yet, it is estimated that the delay between onset and diagnosis of diabetes is approximately 3.5 to 9 years for typical patients given the insidious nature of type 2 diabetes.^8^ As a consequence, up to 20% of individuals already have signs of DR at the time of their type 2 diabetes diagnosis.^9,10^

A recent innovation in ophthalmology imaging has been the incorporation of adaptive optics (AO) into traditional imaging modalities, such as using optical coherence tomography (OCT), a flood illumination ophthalmoscope, or a scanning laser ophthalmoscope (SLO).^11^ AO is a combination of electro-optical and computational methods that help remove optical aberrations in imaging, allowing for greater image resolution than previously possible through traditional imaging modalities.^12^ As a result, AO has provided the ability to directly image and quantify individual photoreceptors.^13^ With no contraindications or associated side effects, AO allows for easy comparison of photoreceptor characteristics between different patient populations.^11^

Previous studies of patients with diabetes that used electroretinograms found significant differences in neuronal cells, including retinal photoreceptors, compared with healthy individuals.^14,15^ If AO-measured photoreceptor outcomes are significantly and reliably different between diabetic populations and healthy populations, AO could potentially be used as a screening modality for the onset of diabetes or DR. To our knowledge, this is the first systematic review to assess for differences in morphological photoreceptor outcomes between individuals with prediabetes or diabetes and healthy controls.

## Methods

### Search Strategy and Eligibility Criteria

A systematic search strategy was used for articles published between January 2000 and June 2023 on the relevant databases Ovid MEDLINE, Embase, and Cochrane Library (Supplemental Table 1). The inclusion criteria consisted of any observational or randomized study that used AO imaging to compare patients with diabetes or prediabetes with healthy controls. Studies that reported 5 or fewer eyes or contained only qualitative data were excluded. This systematic review adhered to the Declaration of Helsinki; neither ethical approval nor informed consent was required. The protocol was prospectively registered in the International Prospective Register of Systematic Review database (No. CRD42023432105).

### Study Selection and Data Collection

The screening process for this systematic review was conducted in 2 separate phases; that is, title and abstract screening followed by full text screening. All screening was performed by 2 independent reviewers (J.Grad, A.H). Any conflicts that arose during the screening process were resolved by an expert, independent third reviewer (N.C).

**Figure 1.**
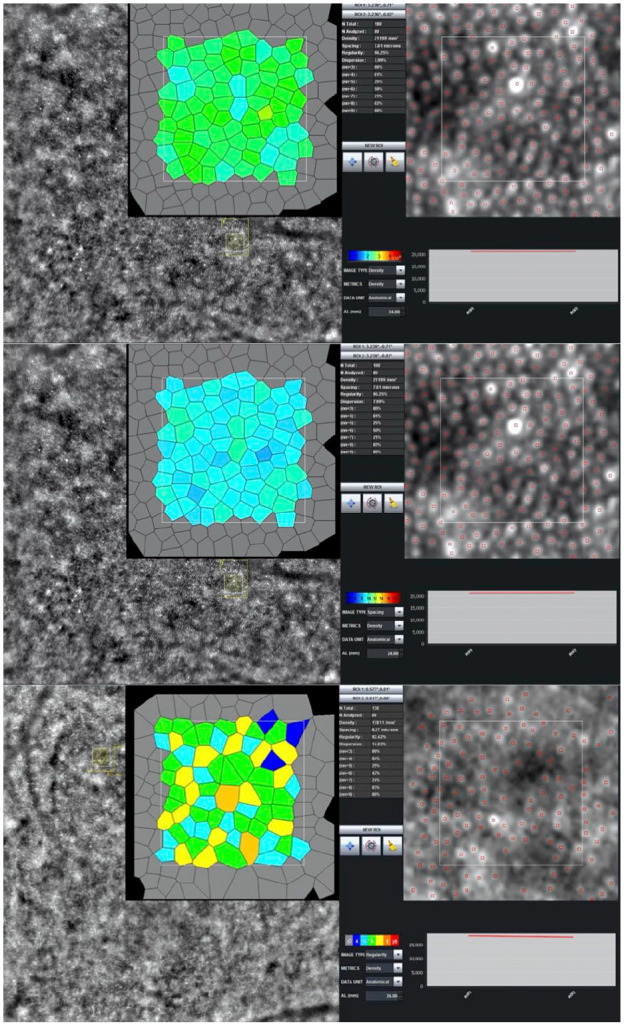
A sample adaptive optics fundus photograph showing image resolution and cone segmentation within a highlighted region of interest.

**Figure 2.**
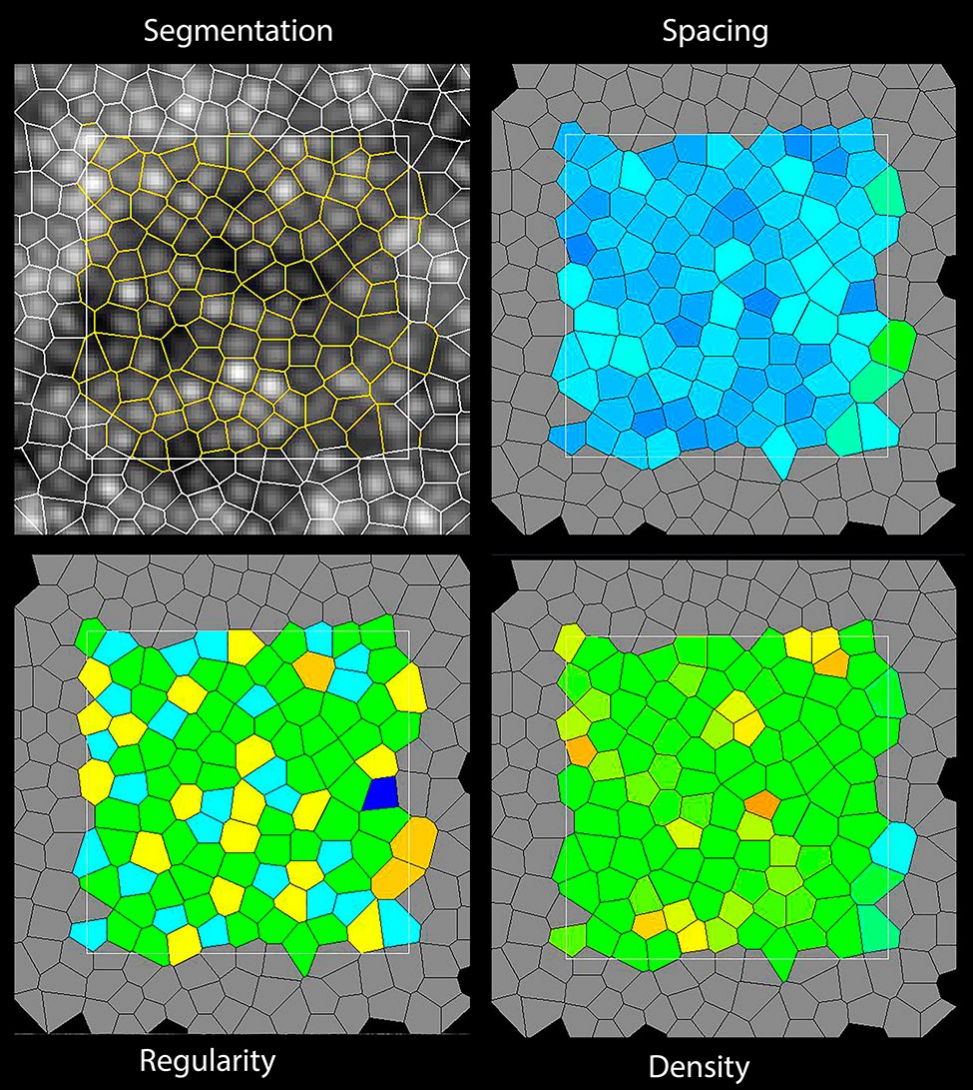
A representative image of morphological photoreceptor outcomes.

The following baseline characteristic data were extracted when available: publication year, study country, study design, number of eyes, age, proportion of men, initial best-corrected visual acuity, type of diabetes included, duration of diabetes, and imaging modality used. This systematic review focused on quantitative data with regard to photoreceptor morphological outcomes. The main expected relevant outcomes were cone density, cone spacing, and cone regularity, all of which can be measured in a variety of ways. Figures 1 and 2 show representative images of AO imaging and relevant photoreceptor parameters. The regions of interest where photoreceptor outcomes were recorded was noted for all included studies. All data were collected and input into Excel software (Microsoft Corp) by 2 reviewers (J.Grad, A.M.).

### Quality Assessment

The quality of the included studies was assessed using the National Heart, Lung, and Blood Institute’s quality assessment tools. This tool was independently completed by 2 reviewers (J.Grad, A.M), and conflicts were resolved by a third reviewer (N.P.)

## Results

**Figure 3.**
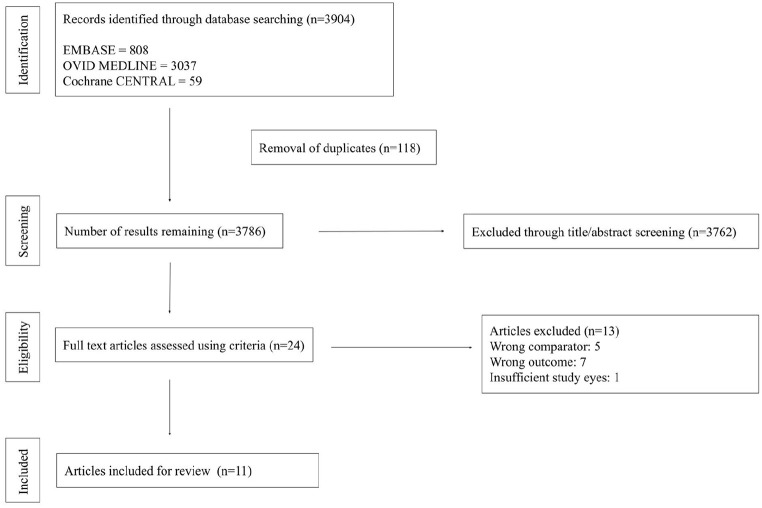
PRISMA flowchart summarizing study screening and selection process.

The systematic search resulted in 3786 unique studies that underwent title and abstract screening. Of those, 24 advanced to full-text screening, and 11 studies of 551 eyes were ultimately included (Figure 3). Three 3 studies used an AO-SLO^16–18^ and 8 studies used an AO-flood illumination ophthalmoscope.^19–26^ Table 1 shows the baseline data of included studies. The quality of all included studies was evaluated using the National Heart, Lung, and Blood Institute’s quality assessment tools (Supplemental Table 2). Table 2 shows the results of the studies.

**Table 1.** Baseline Characteristics of Included Studies.

Author^a^/Year^b^/Country	AO Imaging Modality	Diabetes Type	Comparators (n = Baseline No. of Eyes)	Mean Age (Y) ± SD	Duration Of Diabetes	Male Patients (%)	Mean AL (mm) ± SD	Regions of Interest for Outcome Measurements
Viggiano^19^/2022/ Italy	FIO	Type 1	Mild NPDR (n = 28)	41.7 ± 5.77	21.4 ± 8.34	50		Parafoveal (1–2 mm space around fovea)
			No DR (n = 12)	36.7 ± 5.62	12.3 ± 4.66	58.3		
			Controls (n = 10)	38.1 ± 2.97	N/A	30		
Elsner^16^/2022/USA	SLO	Unclear	Diabetes (n = 10)	54.7 ± 12.8		0	23.0 ± 0.66	Random sampling of 0.9º –7º of eccentricity within temporal retina
			Younger controls (n = 36)	24.4 ± 3.42		0		
			Older controls (n = 10)	56.3 ± 3.71		0		
Ro-Mase^20^/2020/Japan	FIO	Type 2	PDR (n = 10)	49.7 ± 13.3	12.6 ± 10.5	50		Foveal (1 mm space around fovea) & parafoveal (1–2.5 mm space around fovea)
			NPDR (n = 12)	62.5 ± 8.2	13.5 ± 7.2	58.3		
			No DR (n = 4)	53.8 ± 9.2	7.5 ± 7.7	66.7		
			Controls (n = 13)	53.2 ± 9.1	N/A	53.8		
Cristescu^21^/2019/ Romania	FIO	Type 1	No DR (n = 15)	36.4 ± 6.46	19.13 ± 7.47	66.7	23.67 ± 0.82	2º, 3º, & 4º of eccentricity along 4 meridians^c^
			Controls (n = 16)	39 ± 7.75	N/A	62.5	23.93 ± 0.82	
Zaleska-Żmijewska^22^/2019/ Poland	FIO	Unclear	NPDR (n = 36)	49.0 ± 8	24 patients had diabetes for >10 y & 12 between 5 y & 10 y	61.1	23.1 ± 1.1	2º of eccentricity along 4 meridians^c^
			Controls (n = 20)	46.0 ± 10.0	N/A	35	23.1 ± 1.0	
Zaleska-Żmijewska^23^/2017/Poland	FIO	Prediabetes	Prediabetes (n = 12)	52.6 ± 10.0		25	23.78 ± 1.52	3º of eccentricity along 4 meridians^c^
			Controls (n = 22)	42.4 ± 14.3		40.9	24.24 ± 1.45	
Lammer^17^/2016/USA	SLO	Type 1 or 2	PDR (n = 5)			0		Mean 4.3º ± 0.6º of eccentricity along the 4 meridians^c^
			Severe NPDR (n = 6)			0		
			Moderate NPDR (n = 11)			0		
			Mild NPDR (n = 10)			0		
			No DR (n = 11)			0		
			Controls (n = 10)		N/A	0		
			All groups (n = 53)	44 ± 12.8	21.7 ± 12.7	49.1	23.73 ± 0.86	
Soliman^24^/2016/USA	FIO	Type 2	Severe NPDR/PDR (n = 5)	55 ± 10	16 ± 6	80	23.8 ± 1	0º and 2º of eccentricity along 4 meridians^c^
			Moderate NPDR (n = 8)	52 ± 8	18 ± 8	37.5	23.7 ± 1	
			Mild NPDR (n = 7)	50 ± 9	15 ± 10	57.1	23.9 ± 1	
			No DR (n = 9)	53 ± 8	10 ± 3	55.6	23.2 ± 1	
			Controls (n = 20)	55 ± 8	N/A	10	23.4 ± 1	
Lombardo^25^/2016/Italy	FIO	Type 1	NPDR (n = 8)	42.8 ± 9.0	17.9 ± 8.5	37.5		1.5º of eccentricity along 4 meridians^c^
			No DR (n = 8)	37.0 ± 6.6	10.5 ± 2.1	62.5		
			Controls (n = 20)	36.4 ± 8.6	N/A	35		
Tan^18^/2015/ Canada	SLO	Type 1	Diabetes (n = 29)	19.06 ± 3.0	10.69 ± 3.9	62.1	23.3 ± 0.7	5º of horizontal & vertical eccentricity in 4 meridians^c^
			Controls (n = 44)	18.51 ± 3.36	N/A	27.3	23.7 ± 0.8	
Lombardo^26^/2014/ Italy	FIO	Type 1	Diabetes (n = 11)	41.00 ± 6.83	13.64 ± 4.03	45.5	23.80 ± 1.14	230 µm, 350 µm, & 460 µm from fovea along 4 meridians^c^
			Controls (n = 11)	38.50 ± 9.10	N/A	36.7	23.75 ± 0.92	

Abbreviations: AL, axial length; AO, adaptive optics; DR, diabetic retinopathy; FIO, flood illumination ophthalmoscope; N/A, not applicable; NPDR, nonproliferative diabetic retinopathy; PDR, proliferative diabetic retinopathy; SLO, scanning laser ophthalmoscope.

a First author.

b Year of publication.

c The 4 meridians are the superior meridian, inferior meridian, temporal meridian, and nasal meridian.

**Table 2.** Key Findings of Included Studies.

Study	Cone Density	Cone Spacing	Cone Regularity
Viggiano^19^	Cone density was similar between all groups.	Cone spacing was significantly lower in controls compared with those with NPDR (*P* = .002). Cone spacing was similar between no DR and control groups.	Significantly more irregularity was in NPDR eyes relative to control (*P* *≤* .001) and no DR (*P* = .036) eyes. No differences in regularity were observed between control and no DR groups.
Elsner^16^	Most patients with diabetes had cone density <95% CI of healthy controls; however, statistical significance was not assessed.	N/A	N/A
Ro-Mase^20^	Cone density was significantly decreased in the foveal region of patients with PDR vs no DR (*P* = .044). Similar foveal cone densities were observed between the no DR and NPDR groups and the NPDR and PDR groups.	N/A	Cone regularity was similar between all groups in the foveal region. In the parafoveal region, cone regularity was significantly more regular in no DR vs NPDR (*P* = .012) or PDR (*P* = .037) groups, with no significant differences between NPDR and PDR groups.
Cristescu^21^	Cone density was significantly lower in patients with diabetes at all locations (*P* *≤* .05) except at 2 (*P* = .1) and 4 degrees (*P* = .117) of retinal eccentricity along the temporal meridian.	N/A	N/A
Zaleska-Zmijewska^22^	Cone density was significantly lower in all DR groups compared with controls (*P* *≤* .001).	Cone spacing was significantly greater in patients with DR compared with controls (*P* *≤* .001). No significant cone spacing differences were found between patients with mild and moderate NPDR (*P* = .214).	Healthy controls had a significantly more regular cone mosaic compared with DR groups (*P* *≤* .001). No significant differences in regularity were found between patients with mild and moderate NPDR (*P* = .505).
Zaleska-Zmijewska^23^	Cone density was similar between patients with diabetes and prediabetes (*P* = .0928).	The mean cone spacing was similar between patients with diabetes and prediabetes (*P* = .106).	Patients with prediabetes were found to have significantly less regular cone mosaics compared with controls (*P* = .0198).
Lammer^17^	The study found no significant differences in cone density between patients with diabetes compared with controls.	The study found no significant differences in cone spacing between patients with diabetes and controls.	Cone regularity was significantly less regular in diabetic eyes compared with controls (*P* = .04). Cone irregularity increased with DR severity (*P* = .04).
Soliman^24^	Cone density was significantly lower in patients with diabetes compared with the control group (*P* < .001). Cone density was significantly lower in patients with moderate NPDR and severe NPDR/PDR compared with healthy controls (*P* < .001, *P* < .001) and those with no DR (*P* = .038, *P* = .01, respectively). No significant differences were observed in cone density between the control, no DR, and mild NPDR groups.	Cone spacing was found to be greater in moderate and severe NPDR/PDR groups compared with the healthy control (*P* = .003, *P* = .003) and no DR groups (*P* = .012, *P* = .01, respectively). No significant differences were observed in cone spacing between the control, no DR, and mild NPDR groups.	Eyes with moderate NPDR and severe NPDR/PDR had significantly less regular cone mosaics than controls (*P* = .038, *P* = .024, respectively) and individuals with no DR (*P* = .029, *P* = .018, respectively). No significant differences were observed in cone regularity outcomes between the control, no DR, and mild NPDR groups.
Lombardo^25^	Cone density was significantly lower in the NPDR (*P* *≤* .001) and no DR groups (*P* *≤* 0.001) relative to controls.	Mean cone spacing was significantly higher in the NPDR (*P* *≤* .001) and no DR (*P* *≤* .001) groups compared with controls.	Cone regularity was significantly less regular in the NPDR (*P* *≤* .001) and no DR (*P* *≤* .001) groups compared with healthy controls. Similar cone regularity was observed between the NPDR and no DR groups.
Tan^18^	Cone density was not significantly different between individuals with diabetes and controls (*P* = .46).	N/A	N/A
Lombardo^26^	Cone density was significantly lower in patients with diabetes at all 3 measured eccentricities compared with the healthy controls (*P* *≤* .001).	The mean cone spacing was on average 5%–7% larger in patients with diabetes; however, the statistical significance of this finding was not examined.	N/A

Abbreviations: DR, diabetic retinopathy; N/A, not applicable; NPDR, nonproliferative diabetic retinopathy; PDR, proliferative diabetic retinopathy.

### AO-Integrated SLO

The study by Elsner et al^16^ was a prospective cross-sectional study that compared laboratory values of 10 patients with diabetes with published reference values in 36 younger (mean age, 24.4 ± 3.42 years) patients and 10 older patients (mean age, 56.3 ± 3.71 years). The ocular health of the patients with diabetes ranged from no DR to proliferative DR (PDR). The study found that 7 of the 10 patients with diabetes had a cone density lower than 95% CI of the healthy controls; however, statistical significance was not assessed.

A cross-sectional study by Lammer et al^17^ included 43 diabetic eyes and 10 healthy controls. Of the diabetic eyes, 11 had no signs of DR, 10 had mild nonproliferative DR (NPDR), 11 had moderate NPDR, 6 had severe NPDR, and 5 had PDR. Nine patients in these groups had diabetic macular edema (DME). The study found no significant differences in cone density or cone spacing measures between patients with diabetes compared with controls.

Cone regularity was measured using nearest-neighbor distances, which is the average of the minimum distances from the center of a cell to the centers of every other cell in the mosaic, and Voronoi tile analysis, which is a geometric approach that focuses on the number of neighbors for every cone in the mosaic. Nearest-neighbor distances and Voronoi tile analysis were both significantly lower in patients with diabetes than in controls (both *P* = .04). Cone regularity assessed using nearest-neighbor distances was more irregular with increasing DR disease severity (*P* = .04), while no significant association was found with Voronoi tile analysis (*P* = .08). Eyes with DME had significantly lower cone density (*P* = .04), cone spacing (*P* = .03), and cone regularity when assessed with nearest-neighbor distances and Voronoi tile analysis (*P* = .04, *P* = .05) than eyes without DME.

Tan et al^18^ led a cross-sectional study that imaged 73 participants between the ages of 10 years and 25 years. Of these participants, 29 had type 1 diabetes and 44 were healthy controls. The patients showed no signs of DR. The researchers found that cone density did not differ significantly between groups (*P* = .46).

### AO-Integrated Flood Illumination Ophthalmoscopy

A cross-sectional study by Viggiano et al^19^ included 40 patients with type 1 diabetes and 10 healthy age-matched controls. Among the patients with diabetes, 12 showed no signs of DR and 28 showed signs of NPDR. Cone density was similar across all groups. Cone spacing was significantly lower in controls than in those with NPDR (*P* = .002). Conversely, cone spacing was similar between patients with diabetes without DR and patients in the control group. Cone regularity, which was assessed using the heterogeneity packing index, was significantly lower in eyes with NPDR than in control eyes (*P* *≤* .001) and eyes without DR (*P* = .036). No differences were observed between controls and those without DR.

The cross-sectional study by Ro-Mase et al^20^ included 26 patients with type 2 diabetes and 13 healthy controls. Of the patients with diabetes, 4 had no DR, 12 had NPDR, and 10 had PDR. The patients with NPDR were significantly older than those with PDR (*P* = .033), which may have affected the study’s results. Cone density was significantly decreased in the foveal region of patients with PDR compared with those without DR (*P* = .044). Similar foveal cone densities were observed between the no DR group and NPDR group and the NPDR group and PDR group. Cone regularity that was assessed through Voronoi tile analysis was similar among all groups in the foveal region. In the parafoveal region, there was significantly more cone regularity in the parafoveal area in patients with diabetes without DR than in those with NPDR (*P* = .012) or PDR (*P* = .037). No significant differences were observed between the NPDR group and PDR group.

Across sectional study by Cristescu et al^21^ included 15 individuals with type 1 diabetes and 16 healthy controls. None of the patients with diabetes had any signs or a history of DR. The study found a significantly lower cone density in patients with diabetes at all locations (*P* ≤ .05) except at 2 degrees (*P* = .1) and 4 degrees (*P* = .117) of retinal eccentricity along the temporal meridian.

Another cross-sectional study by Zaleska-Żmijewska et al^22^ included 36 patients with DR and 20 healthy controls. The patients with DR were further divided by disease severity, with mild NPDR in 22 patients and moderate NPDR in 14 patients. Cone density was significantly lower in all DR groups than in the control group (*P* ≤ .001). Cone spacing was significantly greater in the DR group than in the control group (*P* < .001). There were no significant differences between patients with mild NPDR and those with moderate NPDR (*P* = .214). Cone packing regularity assessed through Voronoi tile analysis found that healthy controls had a significantly more regular cone mosaic than the patients in the DR groups (*P* ≤ .001) and that there were no significant differences between the mild NPDR group and moderate NPDR group (*P* = .505).

In a case-control study, Zaleska-Żmijewska et al^23^ assessed 12 patients with prediabetes and 22 healthy controls. The criteria used for prediabetes in this study were patients with a hemoglobin A_1c_ level of 5.7% to 6.4%, Cone density was similar between patients with diabetes and those with prediabetes (*P* = .0928). The average cone spacing was similar between the 2 groups (*P* = .106). Voronoi tile analysis found significantly less cone regularity in patients with prediabetes (*P* = .0198).

A cross-sectional study by Soliman et al^24^ included 25 patients (29 eyes) with type 2 diabetes and 10 healthy controls (20 eyes). Of the eyes with diabetes, 9 had no DR, 7 had mild NPDR, 8 had moderate NPDR, and 5 had severe NPDR or PDR. Cone density was significantly lower in patients with diabetes than in the control group (*P* < .001) and in patients with moderate NPDR and severe NPDR/PDR than in the control group (both *P* < .001) and no DR group (*P* = .038 and *P* = .01, respectively). Cone spacing was greater in patients with moderate and severe NPDR/PDR than in the control group (both *P* = .003) and no DR group (*P* = .012 and *P* = .01, respectively). Voronoi tile analysis of cone regularity found that the moderate NPDR group and severe NPDR/PDR groups had significantly less regular cone mosaics than the control group (*P* = .038 and *P* = .024, respectively) and no DR group (*P* = .029, *P* = .018, respectively). No significant differences were observed in cone density, cone spacing, or cone regularity outcomes between the control group, no DR group, and mild NPDR group.

Lombardo et al^25^ performed a cross-sectional study that included 16 patients with type 1 diabetes and 20 healthy, age-matched controls. Of those with diabetes, 8 had no signs of DR and 8 had mild NPDR. Cone density was significantly lower in the NPDR group (*P* ≤ .001) and no DR group (*P* ≤ .001) than in the control group. The mean cone spacing was significantly higher in the NPDR group (*P* ≤ .001) and no DR group (*P* ≤ .001) than in the control group. Voronoi tile analysis found significantly less cone regularity in the NPDR group (*P* ≤ .001) and no DR group (*P* ≤ .001) groups than in the healthy control group and that cone regularity was similar between the NPDR group and no DR group. A logistic regression model demonstrated 100% accuracy in identifying cases of diabetes without DR vs controls when incorporating cone density, spacing, and regularity into the model.

The cross-sectional study by Lombardo et al^26^ included 11 patients with type 1 diabetes and 11 healthy controls. Of those with diabetes, 5 had no signs of DR and 6 had mild NPDR. Cone density was significantly lower in patients with diabetes at all 3 eccentricities than in healthy controls (*P* ≤ .001). The mean cone spacing was approximately 5% to 7% larger in patients with diabetes; however, the statistical significance of this finding was not examined.

## Conclusions

This systematic review summarized studies that used AO to compare morphological photoreceptor outcomes between patients with diabetes or prediabetes and healthy controls. We believe this is the first systematic review to assess morphological photoreceptor outcomes acquired through AO imaging.

Of the 11 included studies that compared patients with diabetes or prediabetes with healthy controls, 9 found at least 1 significant difference,^17,19–26^ 1 noted a difference between groups without analyzing its significance,^16^ and 1 found no significant differences.^18^ Of note, the study that found no significant differences between groups analyzed only cone densities and not cone spacing or regularity.^18^ When examining different photoreceptor measurements, cone regularity was the most consistent in detecting significant differences between healthy populations and those with diabetes. All 7 studies that compared cone regularity outcomes found at least 1 significant difference between the groups with diabetes and the control group.^17,19,20,22–25^

Cone density and cone spacing appear less likely than cone regularity to result in significant differences between patient populations. Of the studies that assessed for statistically significant differences between individuals with diabetes or prediabetes and healthy controls, at least 1 significant difference in cone density^17,19,20,22,24–26^ and spacing^19,22,24,25^ was noted in in 7 of 10 studies^17–26^ and 4 of 6 studies,^17,19,22–25^ respectively. Future AO imaging studies focusing on diabetes or DR should continue to assess patients using all available outcome measures, and particular emphasis should be placed on measuring cone regularity because it appears to be the most sensitive in detecting photoreceptor changes between groups.

This review found differences in morphological photoreceptors between individuals with diabetes and those who are healthy. The retina is a highly metabolic and demanding tissue, with the greatest demand arising from the photoreceptors.^27,28^ Diabetes is known to alter blood flow in both the retina and in the choroid.^29,30^ It is likely that vascular changes observed in DR lead to regions of hypoxia, causing neuronal death and structural rearrangement.^31^ A recent study by Fragiotta et al^32^ of patients with diabetes found that decreases in the perfusion density of the superficial capillary plexus and the choriocapillaris were significantly associated with overlying changes in cone spacing and cone regularity.

Although AO appears to be relatively sensitive in detecting changes in eyes with DR compared with healthy eyes, we noted that AO imaging was not as reliable in detecting differences between healthy individuals and those with diabetes without DR or early DR changes. In the 3 studies that explicitly compared eyes with mild NPDR with eyes with diabetes and no DR, no significant differences were observed in any morphological photoreceptor outcomes.^17,24,25^ Likewise, in the 6 studies that examined patients with diabetes without DR with healthy controls, 3 studies noted at least 1 significant difference in cone morphological outcomes^17,21,25^ and 3 studies found no significant differences between groups.^18,19,24^ However, it is pertinent to highlight that Lombardo et al^25^ were able to differentiate between patients with diabetes without DR and control patients with 100% accuracy using a logistic regression model that incorporated only cone density, spacing, and regularity measurements.

There are some important limitations of this review. First, because of the substantial heterogeneity between studies, we were unable to conduct a meta-analysis that quantitatively assessed the differences between groups. Studies calculated different measurements at various retinal locations, making direct comparisons unreasonable. Furthermore, the heterogeneity arising from studies using differing imaging modalities and protocols was quite substantial and may account for some of the differences in the included studies.

In addition, 7 studies^16–18,21–23,25^ determined regions of interest using degrees of retinal eccentricity from the fovea, a parameter that is dependent on an individual’s ocular axial length.^33,34^ Because axial lengths varied among the included individuals in the study, the calculated regions of interests would not be at a uniform distance from the fovea, even within the same study. The variation in region-of-interest locations is particularly relevant because cone density has been shown to significantly decrease with increasing eccentricity from the fovea.^35^ Notably, every study except for one^25^ reported study the participants’ axial lengths, which helped control the confounding effect of axial length.

Despite the benefits of AO-integrated imaging highlighted by this systematic review, there are key current challenges to widespread adoption in clinical practice, including the cost, the necessity of skilled operators to acquire images, and a lack of normative databases. At present, AO imaging devices are significantly more expensive than many other common imaging devices, likely dissuading clinicians from making the initial investment in an AO device.^12^ In addition, substantial research is still needed to establish normative databases to enable routine use of AO technology in clinical practice.^36^

Although smaller and more operator-friendly commercial devices are becoming available, it appears we are still a handful of years and AO developments away from routine clinical use outside the research setting.^37^ Encouragingly, imaging devices with established use in ophthalmology are beginning to be explored as diagnosis tools and risk prognosticators in diabetes and DR. A recent study found that OCT, a device present in nearly every ophthalmologist’s office, was able to reliably identify individuals with diabetes.^38^ Furthermore, there is an expanding body of literature on the prognostic value of OCT biomarkers, such as intraretinal cysts, hyperreflective foci, the presence of subretinal fluid, and the integrity of individual retinal layers.^39^ OCT angiography is another common imaging modality that is being investigated for its ability to detect early-onset diabetic disease activity and prognosticate the risk for DR advancement through its analysis of microvascular changes.^40^

Overall, this review found that morphological photoreceptor outcomes measured using AO differed between healthy patients and those with diabetes, especially in those with increasing disease severity. Additional research is necessary to confirm the extent to which morphological photoreceptor outcomes differ from healthy individuals, especially in patients with diabetes without DR or mild NPDR. We encourage future research to emphasize using standardized imaging protocols and region of interest locations to allow for more direct comparisons and quantitative syntheses of the literature on this topic.

## Supplemental Material

sj-docx-1-vrd-10.1177_24741264241286682 – Supplemental material for Photoreceptor Characteristics in Diabetic Retinopathy vs Controls Using Adaptive Optics Imaging: Systematic Review Supplemental material, sj-docx-1-vrd-10.1177_24741264241286682 for Photoreceptor Characteristics in Diabetic Retinopathy vs Controls Using Adaptive Optics Imaging: Systematic Review by Justin Grad, Amin Hatamnejad, Niveditha Pattathil, John Golding and Netan Choudhry in Journal of VitreoRetinal Diseases

sj-docx-2-vrd-10.1177_24741264241286682 – Supplemental material for Photoreceptor Characteristics in Diabetic Retinopathy vs Controls Using Adaptive Optics Imaging: Systematic Review Supplemental material, sj-docx-2-vrd-10.1177_24741264241286682 for Photoreceptor Characteristics in Diabetic Retinopathy vs Controls Using Adaptive Optics Imaging: Systematic Review by Justin Grad, Amin Hatamnejad, Niveditha Pattathil, John Golding and Netan Choudhry in Journal of VitreoRetinal Diseases
