# Supplementary material for: Photoreceptor Characteristics in Diabetic Retinopathy vs Controls Using Adaptive Optics Imaging: Systematic Review
Source: J Vitreoretin Dis. 2024 Sep 30:24741264241286682. Online ahead of print. doi: 10.1177/24741264241286682 (PMC11556365; doi:10.1177/24741264241286682)
Supplement: sj-docx-1-vrd-10.1177_24741264241286682 – Supplemental material for Photoreceptor Characteristics in Diabetic Retinopathy vs Controls Using Adaptive Optics Imaging: Systematic Review [file sj-docx-1-vrd-10.1177_24741264241286682.docx]

| **Supplemental Table 1. Systematic Search Strategy** | | |
| --- | --- | --- |
| **OVID Medline Epub Ahead of Print, In-Process & Other Non-Indexed Citations, Ovid MEDLINE(R) Daily and Ovid MEDLINE(R) 1946 to Present** | | |
| 1 | Retinal Cone Photoreceptor Cells/ | 5392 |
| 2 | adaptive opti*.mp. | 3158 |
| 3 | cone cel*.mp. | 830 |
| 4 | cone densi*.mp. [mp=title, book title, abstract, original title, name of substance word, subject heading word, floating sub-heading word, keyword heading word, organism supplementary concept word, protocol supplementary concept word, rare disease supplementary concept word, unique identifier, synonyms, population supplementary concept word, anatomy supplementary concept word] | 426 |
| 5 | exp Cell Count/ | 228315 |
| 6 | 1 or 2 or 3 or 4 or 5 | 236926 |
| 7 | exp Diabetes Mellitus/ | 510116 |
| 8 | exp Diabetic Retinopathy/ | 29683 |
| 9 | exp Prediabetic State/ | 9252 |
| 10 | diabet*.mp. | 848761 |
| 11 | pre?diabet*.mp. | 14762 |
| 12 | 7 or 8 or 9 or 10 or 11 | 851900 |
| 13 | 6 and 12 | 4160 |
| 14 | limit 13 to english language | 3871 |
| 15 | limit 14 to yr="2000 -Current" | 3037 |
| **Embase <1974 to Present>** | | |
| 1 | retina cone/ | 9779 |
| 2 | adaptive opti*.mp. | 3624 |
| 3 | cone densi*.mp. | 770 |
| 4 | exp cell count/ | 523822 |
| 5 | Cone cel*.mp. | 1042 |
| 6 | 1 or 2 or 3 or 4 or 5 | 536903 |
| 7 | exp diabetes mellitus/ | 1222700 |
| 8 | exp diabetic retinopathy/ | 54709 |
| 9 | exp impaired glucose tolerance/ | 37193 |
| 10 | diabet*.mp. | 1452278 |
| 11 | pre?diabet*.mp. | 16796 |
| 12 | 7 or 8 or 9 or 10 or 11 | 1459026 |
| 13 | eye/ | 55092 |
| 14 | diabetic eye disease/ | 326 |
| 15 | eye.mp. | 596107 |
| 16 | 13 or 14 or 15 | 596107 |
| 17 | 6 and 12 and 16 | 808 |
| **Cochrane Central Register of Controlled Trials** | | |
| 1 | adaptive opti*.mp. [mp=title, original title, abstract, floating sub-heading word, mesh headings, heading words, keyword] | 51 |
| 2 | Cone Densi*.mp. [mp=title, original title, abstract, floating sub-heading word, mesh headings, heading words, keyword] | 6 |
| 3 | Cone cel*.mp. [mp=title, original title, abstract, floating sub-heading word, mesh headings, heading words, keyword] | 2 |
| 4 | SLO.mp. [mp=title, original title, abstract, floating sub-heading word, mesh headings, heading words, keyword] | 98 |
| 5 | FIO.mp. [mp=title, original title, abstract, floating sub-heading word, mesh headings, heading words, keyword] | 263 |
| 6 | photorecepto*.mp. [mp=title, original title, abstract, floating sub-heading word, mesh headings, heading words, keyword] | 316 |
| 7 | 1 or 2 or 3 or 4 or 5 or 6 | 713 |
| 8 | diabet*.mp. [mp=title, original title, abstract, floating sub-heading word, mesh headings, heading words, keyword] | 114865 |
| 9 | prediabet*.mp. [mp=title, original title, abstract, floating sub-heading word, mesh headings, heading words, keyword] | 3108 |
| 10 | 8 or 9 | 115485 |
| 11 | 7 and 10 | 59 |
