# Supplementary material for: Photoreceptor Characteristics in Diabetic Retinopathy vs Controls Using Adaptive Optics Imaging: Systematic Review
Source: J Vitreoretin Dis. 2024 Sep 30:24741264241286682. Online ahead of print. doi: 10.1177/24741264241286682 (PMC11556365; doi:10.1177/24741264241286682)
Supplement: sj-docx-2-vrd-10.1177_24741264241286682 – Supplemental material for Photoreceptor Characteristics in Diabetic Retinopathy vs Controls Using Adaptive Optics Imaging: Systematic Review [file sj-docx-2-vrd-10.1177_24741264241286682.docx]

Supplemental Table 2. The quality of all included studies was evaluated using the National Heart, Lung, and Blood Institute’s quality assessment tool

Q1: Was the research question or objective in this paper clearly stated?; Q2: Was the study population clearly specified and defined?; Q3: Were all the subjects selected or recruited from the same or similar populations? Were inclusion and exclusion criteria prespecified and applied uniformly to all participants?; Q4: Was a sample size justification, power description, or variance and effect estimates provided?; Q5: For the analyses in this paper, were the exposure(s) of interest measured prior to the outcome(s) being measured?; Q6: Was the timeframe sufficient so that one could reasonably expect to see an association between exposure and outcome if it existed?; Q7: For exposures that can vary in amount or level, did the study examine different levels of the exposure as related to the outcome? Q8: Were the exposure measures clearly defined, valid, reliable, and implemented consistently across all study participants?; Q9: Was the exposure(s) assessed more than once over time?; Q10: Were the outcome measures clearly defined, valid, reliable, and implemented consistently across all study participants?; Q11: Were the outcome assessors blinded to the exposure status of participants?; Q12: Was loss to follow-up after baseline 20% or less?; Q13: Were key potential confounding variables measured and adjusted statistically for their impact on the relationship between exposure(s) and outcome(s)?

Was the research question or objective in this paper clearly stated?
